# Sanatoria for Consumptives

**Published:** 1899-12

**Authors:** 


**Sanatoria for Consumptives.** By F. RUFENACHT WALTERS, M.D. Pp. xviii., 374. London: Swan Sonnenschein & Co., Lim. 1899.

This book comprises "a critical and detailed description, together with an exposition, of the open-air or hygienic treatment of phthisis," with an introduction by Sir Douglas Powell, who points out that "the chief object of the author is to advocate the establishment in this country of public institutions of the convalescent hospital class, and of sanatoria designed for the reception of cases of tuberculosis from amongst those who are more or less well-to-do." The usefulness of such institutions "extends far beyond the immediate purpose for which they are proposed," inasmuch as "lessons in self-management are learned by those who sojourn for a time in such sanatoria . . . and these persons when they pass again into the general community become centres of instruction in domestic hygiene." The author remarks that "up to recent times England had probably done more to reduce phthisical mortality than any other country; but she is now being outstripped in the race by several others" where "public opinion . . . both lay and medical, is overwhelmingly in favour of the sanatorium system."

This is one of the first attempts to present a general review of the subject in English; the first 82 pages give a general *résumé* of considerations regarding climates, sites for sanatoria, construction, cleansing, disinfection, diet, and general management. An enquirer naturally turns for information to the chapter on results, and it is claimed that the percentage of improvement from sanatorium treatment in different parts of the world varies from 50 to 90 as compared with only 20 to 30 in the Brompton Hospital. It is very difficult to draw any conclusions from statistics compiled by various persons adopting different standards, in a disease subject to so much spontaneous variation as phthisis; but the general average shows a well-marked difference in favour of sanatorium rather than any kind of climatic treatment.

A review of the various sanatoria of the world occupies some 280 pages: these have been compiled with care, and appear tc be fairly accurate. It is shown that there is a great need foı the increase and extension of such institutions in this country, inasmuch as "Professor v. Leyden estimates that if each consumptive has three months' treatment, there should be one bed for every 1000 inhabitants."

The author concludes his interesting survey with the following paragraph:—"The establishment of various societies for the suppression of tuberculosis, one of them under most distinguished patronage, is but one of many signs that Great Britain is at last waking from her satisfied slumbers, and preparing to again take her place in the van of the nations. Our country's sanitary past has been great and fruitful; and there is every reason to hope that with growing consciousness of the possibility of destroying this dread scourge of humanity, by the abolition of town smoke, the improvement of our dwellings, the better ventilation of rooms and streets, the admission of sunshine into our midst, the inculcation of more rational habits of life, the destruction of sputa, the erection of sanatoria, and in many other ways, she will gradually prepare for herself a still more great and glorious future."

The bibliography extends to nine closely printed pages.

